# Health care utilization, prognosis and outcomes of vestibular disease in primary care settings: systematic review

**DOI:** 10.1007/s00415-015-7913-2

**Published:** 2016-04-15

**Authors:** Eva Grill, Mathias Penger, Erna Kentala

**Affiliations:** Institute for Medical Information Processing, Biometrics and Epidemiology, Ludwig-Maximilians-Universität München, Marchioninistraße 17, 81377 Munich, Germany; German Center for Vertigo and Balance Disorders, Ludwig-Maximilians-Universität München, Munich, Germany; Department of Otorhinolaryngology, Helsinki, University Central Hospital, Helsinki, Finland

**Keywords:** Vertigo, Dizziness, Primary care, Epidemiology, Systematic review

## Abstract

Vertigo and dizziness are frequent complaints in primary care that lead to extensive health care utilization. The objective of this systematic review was to examine health care of patients with vertigo and dizziness in primary care settings. Specifically, we wanted to characterize health care utilization, therapeutic and referral behaviour and to examine the outcomes associated with this. A search of the MEDLINE and EMBASE databases was carried out in May 2015 using the search terms ‘vertigo’ or ‘dizziness’ or ‘vestibular and primary care’ to identify suitable studies. We included all studies that were published in the last 10 years in English with the primary diagnoses of vertigo, dizziness and/or vestibular disease. We excluded drug evaluation studies and reports of adverse drug reactions. Data were extracted and appraised by two independent reviewers; 16 studies with a total of 2828 patients were included. Mean age of patients ranged from 45 to 79 with five studies in older adults aged 65 or older. There were considerable variations in diagnostic criteria, referral and therapy while the included studies failed to show significant improvement of patient-reported outcomes. Studies are needed to investigate current practice of care across countries and health systems in a systematic way and to test primary care-based education and training interventions that improve outcomes.

## Introduction

With a high lifetime prevalence [1] and high burden of disease [2], vertigo and dizziness can be severely disabling because of its high impact on daily life [3]. Psychiatric comorbidities such as anxiety, depression, panic disorders, and specific phobias such as agoraphobia or acrophobia may account for avoidance behavior, increased disability [4], and increased health care utilization [5]. Finally, vertigo and dizziness are specific and important risk factors for falls and injuries, especially in the aged [6].

Almost 45 % of outpatients with dizziness and vertigo are primarily seen and treated by a primary care physician (PCP) [7] who is often without specific neuro-otological expertise for the diagnosis and management of vestibular disorders. Management in primary care seems to be difficult because dizziness as a symptom is difficult to describe and to standardize [8]. Also, PCPs might reasonably want to exclude potentially life threatening diseases and utilize all possible diagnostic options to avoid litigation. There is evidence that PCPs are referring such “red flag” cases correctly. However, they failed to refer patients to the specialist when referral would have been appropriate [9].

Most instances of vertigo and dizziness are manageable [10–12]. Peripheral vestibular disorders are frequent causes for dizziness and vertigo; benign paroxysmal positioning vertigo (BPPV) is the most frequent form of peripheral vestibular disorders with a lifetime prevalence of 2 % in the general population [13]. Other, less-frequent peripheral forms of vestibular disorders include Menière’s disease and vestibular neuritis. Central vestibular forms of vertigo include cerebrovascular diseases, brain stem and cerebellar lesions, infections, and vestibular migraine. In aged adults, the ageing of vestibular and proprioceptive systems and, most notably, medication are potential risk factors for vertigo and dizziness.

Inappropriate management of patients with the cardinal symptoms of vertigo and dizziness may lead to chronicity, activity limitations [14] and considerable economic impact [2]. Yet studies conducted from the retrospective perspective of specialized tertiary care centers found considerable under-and misdiagnosis and irrational treatment and management practices in primary care [9, 15, 16]. Since vertigo and dizziness are frequent complaints that lead to extensive health care utilization, and since most patients are likely to rely on PCPs for the management of their complaints, it is important to know more about typical management patterns and referral practices as well as about prognosis and outcomes of dizzy patients in primary care.

Objective of this systematic review was to examine health care of patients with vertigo and dizziness in primary care settings. Specifically, we wanted to characterize health care utilization, therapeutic and referral behaviour and to examine the outcomes associated with this.

## Methods

### Data source

A search of the MEDLINE and EMBASE databases via OVID was carried out in May 2015 using the search terms ‘vertigo’ or ‘dizziness’ or ‘vestibular and primary care’ to identify suitable studies. Additionally, we used the sensitive/broad search filters for outcome assessment proposed by the Health Services Research Queries for MEDLINE [54]. Furthermore, we searched grey literature and checked the references of the included studies. We included all studies that were published in the last 10 years in English with the primary diagnoses of vertigo, dizziness and/or vestibular disease. We excluded drug evaluation studies and reports of adverse drug reactions. Since a pilot search revealed that the number of studies on vertigo in primary care is very limited we included all study designs and types with the exception of tutorials, case reports and case series with *n* < 10. Intervention studies were eligible for inclusion if there was a control arm receiving usual care, and only the results of usual care controls were reported. To ensure the quality of the search strategy, all search strategies were pilot-tested on their ability to find abstracts which were previously identified as relevant.

### Data extraction and analysis

Studies were independently screened for inclusion criteria by two reviewers (EK and EG) based on title and abstract. The selected publications were subsequently reviewed based on full text. Agreement on the criteria for selecting publications had to be reached by consensus. In case of disagreement during the selection process, a third reviewer made the final decision.

We developed a data extraction sheet and pre-tested the sheet on five randomly chosen included studies. One review author extracted the data; the second author checked the extracted data.

## Results

**Fig. 1:**
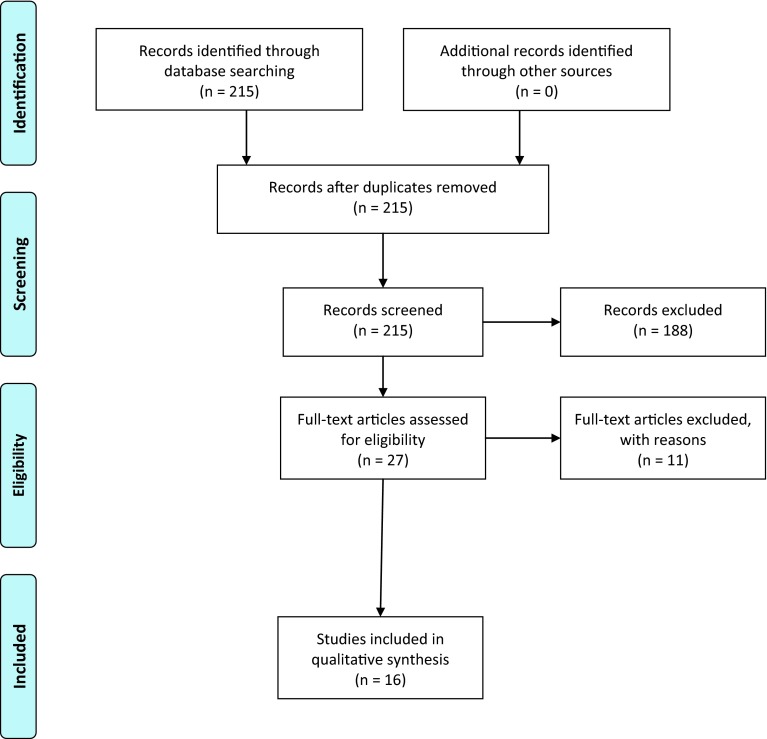
PRISMA flow diagram

We identified 215 records through database searching. One hundred and eighty-eight records could be excluded by screening of the abstracts, leaving 27 full text articles for eligibility assessment. Eleven full texts were excluded because they did not meet inclusion criteria, leaving 16 publications for qualitative review. Of these 16 publications, two reported results from the same cohort study from the Netherlands [17, 18], and two reported results from the same cohort study from Germany [19, 20]. We decided to include all four publications into the qualitative review because they reported different aspects; two gave results of the baseline survey [18, 19], two gave results of the follow-up [17, 20]. Figure 1 shows the PRISMA flow diagram for inclusion. Table 1 shows the characteristics of the included studies.

**Table 1:** Study populations and characteristics of the included studies

#	References	Publication title	Age range	Study design	*N*	Duration of follow-up (months)	Mean age	% female	Number of practices	Country
1	Dros [17]	Functional prognosis of dizziness in older adults in primary care: a prospective cohort study	65–95	Cohort	385, follow-up of #9	6	79	74	24	Netherlands
2	Ekvall Hansson[43]	Benign paroxysmal positional vertigo among elderly patients in primary health care	65–94	Cross-sectional	38	NA	NR	65.8	NR	Sweden
3	Garrigues [21]	Epidemiological aspects of vertigo in the general population of the Autonomic Region of Valencia, Spain	10+	Cohort	191	12	56 (17.6)	68.6	6	Spain
4	Hansson [22]	Balance performance and self-perceived handicap among dizzy patients in primary health care	22–90	Cross-sectional	119	NA	61	61.3	1	Sweden
5	Hansson [30]	Falls among dizzy patients in primary healthcare: an intervention study with control group	65+	Cohort	27	12	NR	NR	6	Sweden
6	Kruschinski [19]	A three-group comparison of acute-onset dizzy, long-term dizzy and non-dizzy older patients in primary care	65–95	Cross-sectional	Acute: 69 Chronic: 86 Baseline of #13	NA	NR	NR	20	Germany
7	Leong [23]	Primary assessment of the vertiginous patient at a pre-ENT balance clinic	Adults	Cross-sectional	102	NA	NR	NR	1	United Kingdom
8	Lin [24]	Otologic diagnoses in the elderly: current utilization and predicted workload increase	65+	Medical claims data	4,480,000	NA	77 (0.7)	63	NA	USA
9	Maarsingh [18]	Causes of persistent dizziness in elderly patients in primary care	65+	Cross-sectional	417, baseline of #1	NA	79	74	24	Netherlands
10	Nazareth [25]	Patterns of presentations of dizziness in primary care–a cross-sectional cluster analysis study	16–62	Cross-sectional	442	NA	NR	NR	2	United Kingdom
11	Neuhauser [2]	Burden of dizziness and vertigo in the community	18–79	Cross-sectional	1003	NA	44.9 (16.6)	65.4 %	NA	Germany
12	Polensek [26]	Screening for vestibular disorders: a study of clinicians’ compliance with recommended practices	Adults	Cross-sectional	157	6	60 (13.6)	11.5	1	USA
13	Sczepanek [20]	Newly diagnosed incident dizziness of older patients: a follow-up study in primary care	65–95	Cohort	66, follow-up of acute cases of #6	6	76 (6.5)	69.6	21	Germany
14	Skoien [31]	Occupational disability caused by dizziness and vertigo: a register-based prospective study	16–62	Medical claims data	1018	NA	NR	68.2	NA	Norway
15	Tschan [27]	Persistence of symptoms in primary somatoform vertigo and dizziness: a disorder “lost” in health care?	Adults	Cohort	65	36	48 (11.88)	46.2	NR	Germany
16	Yardley [28]	Clinical and cost effectiveness of booklet based vestibular rehabilitation for chronic dizziness in primary care: single blind, parallel group, pragmatic, randomised controlled trial	18+	Cohort (RCT)	112	12	58 (15.8)	75	35	United Kingdom

*NA* not applicable, *NR* not reported

Because of the heterogeneity of the included studies we did not appraise their quality systematically.

## Methods

Six of the studies finally selected were cohort studies including one randomized controlled trial and one controlled trial. Eight studies were cross-sectional studies with two reporting baseline results from cohorts as mentioned above. Two studies were analyses of medical claims data or registries. Follow-up in cohort studies ranged between 6 and 12 months. One study reported data from two follow-up examinations [20]; all other cohort studies had one follow-up.

### Participants

A total of 2828 patients were included in all individual studies, not counting cases from country-wide registries. Of those from individual studies, 2433 were of cross-sectional studies, 707 of cohort studies. 139 patients were controls from controlled trials. Mean age of patients ranged from 45 to 79 with 5 studies in older adults aged 65 or older and 9 studies without clear age restrictions with the age of included participants ranging from 10 to 95. Percentage of female patients ranged from 46 to 74 % with one outlier from a veterans’ primary care center (11 % women). Study populations were from Europe or from the US. Diagnoses were mostly unspecific dizziness and vertigo with few studies reporting clear diagnostic criteria for e.g. benign paroxysmal positional vertigo or peripheral vestibular disease. Table 2 provides the diagnostic criteria mentioned in the included studies.

**Table 2:** Description and verification of diagnoses of all included studies

#	References	Diagnoses	Description of verification in full text
1	Dros [17]	Dizziness	A giddy or rotational sensation, a feeling of imbalance, light-headedness, and a sensation of impending faint as reported by consulting patient
2	Ekvall Hansson [43]	Multisensory dizziness with age as one factor; BPPV; vestibular neuronitis, dizziness of unspecific origin	Physical examination including the Hallpike maneuver
3	Garrigues [21]	Vertigo crisis	Illusion of unequivocal rotary movement
4	Hansson [22]	BPPV Phobic postural vertigo cervical/whiplash associated disorder dizziness	Standardized assessment, Dix-Hallpike manoeuvre
5	Hansson [30]	Multisensory dizziness	ICD-10 Code: R42
6	Kruschinski [19]	Chronic/acute dizziness	A sensation of dizziness
7	Leong [23]	BPPV, dizziness, vertigo	NR
8	Lin [24]	Sensorineural hearing loss, tinnitus, Meniere disease, vestibular neuritis, benign paroxysmal positional vertigo [BPPV], and vertigo	ICD-9 codes
9	Maarsingh [18]	Dizziness	Giddy or rotational sensation, a loss of balance, a faint feeling, light-headedness, instability or unsteadiness, a tendency to fall, or a feeling of everything turning black
10	Nazareth [25]	Vertigo, presyncope, disequilibrium, dizziness	True vertigo (i.e., a sensation that things/oneself are moving, spinning, or rocking about), presyncope (feeling of being faint or losing consciousness), disequilibrium (feeling unsteady or off-balance or about to fall or veer to one side), and other types of dizziness (e.g., giddiness, light headedness, or wooziness)
11	Neuhauser [2]	Vestibular disease, dizziness, vertigo	Screening question, “Did you ever experience moderate or severe dizziness or vertigo?” neurotologic interview
12	Polensek [26]	Vestibular impairment, dizziness, vertigo	Dizziness (ICD-9 code 780.4) or any form of vestibular impairment (ICD-9 code 386.0 through 386.9)
13	Sczepanek [20]	Dizziness	A sensation of dizziness
14	Skoien [31]	Dizziness, vertigo	International classification of primary care, H82 (vertiginous syndrome), N17 (vertigo/dizziness)
15	Tschan [27]	Psychogenic vertigo	Clinical neurological examination; a neuro-orthoptic analysis; neurophysiological vestibular laboratory testing including an electro-oculography with caloric irrigation, measurements of the subjective visual vertical, and determination of ocular torsion by fundus photographs, psychometric test battery measuring dizziness-related somatic and mental symptoms. Structured Clinical Interview for Diagnostic and Statistical Manual of Mental Disorders, Fourth Edition (DSM-IV), Axis I disorders
16	Yardley [28]	Chronic dizziness not attributable to non-vestibular causes	Search of computerised patient records search terms: vertigo; dizziness; Meniere’s disease; balance problems; vestibular; prochlorperazine; cinnarizine; betahistine; diuretics (see [44])

*NR* not reported

*ICD-10* international classification of disease, tenth revision

*ICD-9* international classification of disease, ninth revision

## Results of individual studies

### Consultations, referrals and therapy

Ten studies reported data on health care utilization and consultation and referral patterns [2, 20–28]. Lin et al. estimated a total of 1.49 Mio consultations for otologic diagnoses per year for the US population aged 65 and older (own calculations), with a rate of 6 consultations per 1000 aged inhabitants per year for BPPV, 8 per 1000 for Menière’s Disease and 7 per 1000 for vestibular neuritis [24]. For the total population in Spain Garrigues reported that 17.8 individuals per 1000 inhabitants per year had at least one consultation for vertigo, 7.6 per 1000 for acute incident vertigo [21]. Seventeen percent of all adults in Germany had had at least one medical consultation in life for dizziness or vertigo [2]. Of patients with dizziness and vertigo, 60 to 80 % contacted the PCP for treatment in UK [25, 28], in Germany 58 % had at least one medical consultation, also predominantly in primary care, [2]. Likewise, in the US 55 % of patients with dizziness were initially seen by the PCP [26]. Depending on health system and availability physicians from other medical specialities were also consulted with PCPs, neurologists, otorhinolaryngologists and orthopaedists being the most frequently mentioned. Previous hospitalization was reported by 2 % of all German adults [2].

Despite unresolved diagnosis, only 22 % of the patients seen by a PCP in the US veterans’ health service were referred to specialists [26]. In contrast, German PCPs referred 48 % of their dizzy patients to at least one specialist. In 18 % of the cases the specialist’s diagnosis differed from the PCP’s [20]. Also, 46 % of the patients with a confirmed diagnosis of psychosomatic vertigo were nevertheless referred to specialists for further clinical examination and therapy [27]. Two percent of patients initially seen by a German PCP were sent to hospital [20]. Individuals with chronic dizziness rated the physician’s empathy concerning their complaints significantly lower than individuals with acute incident dizziness [20]. For patients with BPPV, median waiting time between referral and effective management was 22 to 27 weeks in the UK [23].

Of those patients seen by a PCP in the UK, 90 % received medication, 40 % physiotherapy, 10 % psychotherapy [25]. Likewise, 58 % of patients with confirmed vestibular causes of dizziness received medication [28]. Even though having received a recommendation, 20 % of the patients with psychosomatic vertigo did not receive psychotherapy, and 20 % received less than 12 sessions of outpatient psychotherapy [27].

### Outcomes and prognosis

As shown in Table 3, only seven studies examined patient-reported outcomes, five of them longitudinally. Most frequently reported outcome was dizziness-specific functioning as operationalized by the Dizziness Handicap Inventory (DHI) [29] or the Vestibular Handicap Questionnaire (VHQ) [17–20, 22, 27, 28, 30]. Four studies examined generic health-related quality of life using four different measures [2, 19, 20, 27, 28]. Two studies reported vertigo-related symptoms using the Vertigo Symptom Scale (VSS) [27, 28]. One study examined the rates of adults with vertigo obtaining disability pension [31]. Generic activities of daily living were examined in one study [19, 20]. One study reported costs and cost per quality adjusted life years [28]. There were no statistically significant or clinically relevant changes in specific functioning during follow-up time. Mean score of the DHI of the included patients was mostly above the threshold of 26 indicating moderate to severe impairment. Score changes failed to reach the minimal clinically relevant difference of 12 [32]. Physical quality of life and vertigo-related symptoms improved significantly in one study in patients with confirmed psychogenic vertigo [27].

**Table 3:** Outcomes measured in included studies

References	Dizziness-specific functioning T1/T2/T3	Quality of life	Symptoms	Other
Dros [17]	DHI (median) 34/24/– Mean change 7.5 ± 19.2	NR	NR	NR
Hansson [30]	DHI 36(22)/33/35 Mean change –/−3/+2	NR	NR	NR
Maarsingh [18]	DHI 36.3	NR	NR	NR
Neuhauser [2]	NR	SF-8, values NR	NR	NR
Sczepanek [20]	DHI: 26.68/22.95/24.32	SF-12: 47.03/49.41/48.64	NR	GDS: 3.19/3.08/2.63 ADL: 39.63/39.72/3953
Tschan 2013 [27]	VHQ 48.70 (19.76)/39.49 (24.46)/–	*PHC (SF-36) 40.54 (10.31)/43.76 (12.19) MHC (SF-36) 40.73 (12.20)/42.57 (13.91)	*VER (VSS-VER) 0.93 (0.50)/0.77 (0.60) Somatic AA (VSS-AA) 1.31 (0.74)/1.31 (0.89)	GSI (SCL-90-R) 0.71 (0.46)/0.63 (0.44)
Yardley [28]	DHI 32.9 (18.4)/28.2 (18.7)/29.2 (18.8)	EQ-5D 0.79 (0.22)/0.79 (0.27)/0.79 (0.26)	VSS short form 13.8 (10.7)/10.5 (8.7)/11.0 (8.7)	Patients reporting improvement (n) 40 of 107 (37 %)/47/99 (47 %)

All numbers are mean (SD) if not stated otherwise. Score values are reported for baseline/first follow-up/second follow-up if available

*NR* not reported, *DHI* dizziness handicap inventory [29], *VHQ* vertigo handicap questionnaire [45], *SF-8,-12, -36* 8-, 12-, 36-item short-form health survey [46–48], *PHC* physical health component, *MHC* mental health component, *EQ-5D* EuroQoL [49], *VSS-VER -AA* vertigo symptom scale - vertigo and related symptoms, -somatic anxiety and autonomic arousal [50], *GDS* geriatric depression scale [51], *SCL-90-R (GSI)* symptom checklist 90 (global severity index) [52], *ADL* activities of daily living [53]

* Difference significant

## Discussion

This systematic review of the recent literature suggests that health care of patients with vertigo and dizziness in primary care settings is still suboptimal. The examined studies failed to show significant improvement of patient-reported outcomes while there were considerable variations in referral and therapy.

Our review suggests that there is a scarcity of studies, specifically of longitudinal studies investigating the processes and outcomes of usual care of vertigo and dizziness in primary care, and of controlled trials testing the implementation of improved care options. The included studies confirm that, regardless of country and health system, about 2 % of the adult population per year sees a physician because of vertigo; among those consultations, PCPs are seen predominantly. These findings from the last 10 years compare well with an earlier study that reported that nearly 45 % of dizzy outpatients are first seen by the general practitioner, and that vertigo and dizziness constitute frequent reasons to see the PCP [7].

Regarding diagnostic criteria, the studies examined here present a large variety of definitions for vertigo and dizziness including the rather superficial coding as it is found in medical claims data. This matches the observation that unclear dizziness often remains the main primary diagnosis and is not further classified into the more specific categories of central or peripheral origin. Especially, BPPV, multisensory dizziness, and vestibular migraine are under-diagnosed by referring physicians [15]. It is noteworthy that diagnostic work-up might be difficult even for specialists due to somehow vague diagnostic criteria and their repetitive change. In a study comparing physicians’ decision making for dizzy patients in an ENT clinic to a neurotologic expert system, the specialists were able to solve 69 % of the cases while the expert system solved 65 % [33]. Ultimately, incomplete diagnostic work-up will lead to unsuccessful therapy attempts and eventually to chronification.

In a recent study from the US, dizziness and vertigo were among the most frequently referred neurological symptoms [34]. There are several possible reasons why the speciality referral rate by PCPs exceeded this 14 % referral rate for neurological symptoms. Vertigo and dizziness are potentially difficult to diagnose for the PCP, thus the amount of complexity of diagnosis and therapy may be one primary reason for referral. With the exception of BPPV, verification of a causative lesion needs sophisticated testing, and the PCP might want to exclude potential life-threatening diseases, e.g. by imaging. Nevertheless, in the majority of cases diagnostic imaging procedures such as magnetic resonance imaging of the head and neck fail to identify the cause of dizziness [35]. Another reason for frequent referral might be that PCPs’ workload precludes detailed investigations. This does not, however, explain why patients with established psychosomatic vertigo are referred for further diagnostic procedures, or why the appropriate treatment, in this case, psychotherapy, is withheld. A recent review of the literature showed that, despite large variation, PCPs’ referral rates do not always reflect optimal care decisions [36]. One major drawback of referral is the potentially long waiting time until appointment with the specialist as reported by a study from the UK [23]. Many instances of peripheral disease might resolve spontaneously within that time or fluctuate. This can affect interpretation of the preliminary findings but also speed chronification and the development of secondary psychosomatic disease.

Regarding treatment, the high percentage of patients receiving medication merits mentioning. Although the use of vestibular suppressants can be indicated in an acute stage of disease, they may inhibit vestibular compensation and are therefore largely inappropriate, e.g. in BPPV [13]. Other therapies with known efficacy were hardly reported; namely vestibular rehabilitation and involvement of physical therapists were underrepresented.

In line with earlier work there was a multitude of different and hardly comparable outcome measures from the domains of functioning, quality of life and symptom severity [37–39]. It is interesting to note that none of the studies investigating mid- and long-term outcomes under usual care conditions could show significant or clinically relevant improvement in functioning. For the dizzy patient, this prospect is hardly satisfying because many causes of vertigo and dizziness are manageable in theory, and have a benign prognosis. To give an example, in a recent observational study with 2 years’ follow-up, patients diagnosed and treated in a specialized tertiary care clinic improved significantly with a mean reduction of the DHI score of 14 points [40]. Favorable change could be observed for all disease entities, but the difference was largest for patients with BPPV. Specifically because BPPV is well-treatable [41] it should primarily be managed in primary care.

We are aware that this systematic review of the literature has several limitations. Although a formal quality appraisal was hardly feasible because of study heterogeneity we noted considerable discrepancies in the included studies regarding methodological rigorousness. In general, sample sizes were small resulting in high variance of the reported prevalences and effect estimates. The small sample size may be partly explained by the recruiting process. The ability of including a representative sample of patients through their PCP will vary depending on the country-specific health system. Research will be easy in countries with a well-developed PCP system with formalized referrals where the PCP acts as a gate-keeper for other specialities, and it may be less straightforward in health systems where community-based specialists compete with PCPs as primary access point for patients [42]. The same applies for varying referral rates. The low number of high-quality studies is surprising but underlines the low awareness of vertigo and dizziness as health problems that are relevant not only to the specialist but rather to daily routine of the PCP [2].

In conclusion, diagnosis and management of vertigo and dizziness needs to be streamlined for primary care. Practical algorithms should be developed and implemented that take into account time and resource restrictions of PCPs, but give pragmatic instructions how to proceed with dizzy patients and to make correct referral and treatment decisions so that those with manageable disease will receive timely treatment, and those who are in risk for serious illness or benefit from specialist consultation will be forwarded to tertiary care. Close cooperation with specialists and other health professions should be part of the routine. Studies are needed to investigate current practice of care across countries and health systems in a systematic way and to test PCP-based education and training interventions that improve patient-reported outcomes.
